# The current status of various preclinical therapeutic approaches for tendon repair

**DOI:** 10.1080/07853890.2024.2337871

**Published:** 2024-05-13

**Authors:** Wenqing Liang, Chao Zhou, Yongjun Deng, Lifeng Fu, Jiayi Zhao, Hengguo Long, Wenyi Ming, Jinxiang Shang, Bin Zeng

**Affiliations:** a Department of Orthopedics, Zhoushan Hospital of Traditional Chinese Medicine Affiliated to Zhejiang Chinese Medical University, Zhoushan, China; b Department of Orthopedics, Zhoushan Guanghua Hospital, Zhoushan, China; c Department of Orthopedics, Affiliated Hospital of Shaoxing University, Shaoxing, China; d Department of Orthopedics, Shaoxing City Keqiao District Hospital of Traditional Chinese Medicine, Shaoxing, China

**Keywords:** Tendon repair, recent advances, therapeutic approaches, preclinical status

## Abstract

Tendons are fibroblastic structures that link muscle and bone. There are two kinds of tendon injuries, including acute and chronic. Each form of injury or deterioration can result in significant pain and loss of tendon function. The recovery of tendon damage is a complex and time-consuming recovery process. Depending on the anatomical location of the tendon tissue, the clinical outcomes are not the same. The healing of the wound process is divided into three stages that overlap: inflammation, proliferation, and tissue remodeling. Furthermore, the curing tendon has a high re-tear rate. Faced with the challenges, tendon injury management is still a clinical issue that must be resolved as soon as possible. Several newer directions and breakthroughs in tendon recovery have emerged in recent years. This article describes tendon injury and summarizes recent advances in tendon recovery, along with stem cell therapy, gene therapy, Platelet-rich plasma remedy, growth factors, drug treatment, and tissue engineering. Despite the recent fast-growing research in tendon recovery treatment, still, none of them translated to the clinical setting. This review provides a detailed overview of tendon injuries and potential preclinical approaches for treating tendon injuries.

## Introduction

Tendons are highly resilient to mechanical stresses and can transmit electrical signals, distributing, and controlling the forces imposed by muscles on the attached tissues. Tendons maintain posture or create movement in this manner [1–3]. Tendons connect bones to muscles, have a strong confrontation to tearing as well as tensile strength, and have an essential role in the stability of bone movement. In reality, however, tendons, which are made up of cells and similar clusters of collagen fibers, are frequently affected and even burst [2]. The yearly prevalence rate of ligament and tendon injuries (TIs) is approximately 18 patients per 100,000 patient population, with a higher incidence rate in sports athletics [4]. Because of a deficiency of cells and growing hormones, tendon recovery is sluggish and of poor quality [5,6].

Tendons are organized hierarchically [7]. Triple-helical type I collagen molecules sequentially form fibers, aggregates, and musculotendinous units. The most prevalent component of the extracellular matrix (ECM) throughout all soft tissues, containing tendons, is type I collagen. The unique structure of the tendon relies on the similar structuring of collagen fibrils of type I rather than the appearance of type I collagen [7,8]. The process by which collagen type I fibers drive distinct spatial configurations in tendons is poorly understood now. In tendon tissue, growth factors (GFs), for instance, transforming growth factors (TGF) and fibroblast growth factors (FGFs), are advantageous for enhancing the appearance of collagen and promoting collagen production [9–11]. Mechanical forces are also employed in the manufacture of type I collagen in tendons: increasing load results in enhanced collagen substance in tendons, and decreased load results in decreased collagen material in the tendons [1,12].

Densely packed collagen fibers in the ECM are deliberated by the fundamental tendon force-transfer unit [1]. A range of ECM components, including collagen, elastin, and glycoprotein, contribute to the formation of collagen type I [13]. Human tendons rupture at 8% strain and undergo plastic distortion at 4% strain [14].

TI is the most prevalent musculoskeletal problem for which people seek medical treatment, according to prior research [15,16]. Before the age of 45, Achilles tendinopathy (AT) affects one out of every 10 persons and one out of every two runners [17]. TI may be triggered by either a rapid break or a persistent condition known as tendinopathy [2,3].

**Figure 1.**
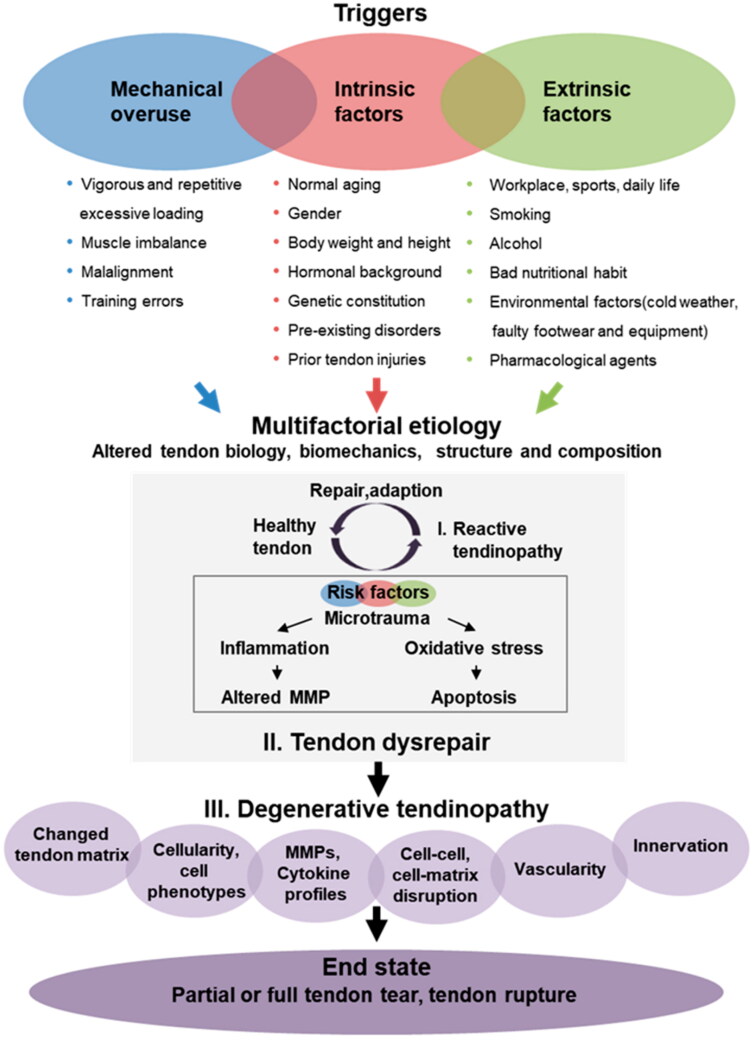
Diagram illustrating the pathophysiology of tendinopathy. It is theorized that a variety of risk factors, containing mechanical abuse along with intrinsic and extrinsic variables, might continuously activate the onset of tendinopathy. First, these risk factors impede adequate tendon renovation, resulting in primary reactive tendinopathy, which still can mend. In addition, the accumulation and growth of risk factors result in tendon damage, which ultimately leads to tendon degeneration. Inadequate tendon function and load capacity ultimately result in tendinopathy, which causes tendon ripping or rupture [22].

Tendon ruptures are often triggered by acute mechanical stress [2,3]. Chronic tendon damage, also known as tendinopathy, is the clinical signs of injured tendons, which include discomfort, localized tendon soreness, diminished strength, and decreased activity. In contrast to partial or total tendon tears, there is no macroscopic tear seen with tendinopathy. Tendinopathy is characterized by the following histological characteristics: disorganized collagen fibers, an upsurge in the content of glycosaminoglycans and proteoglycans, an upsurge in non-collagenous ECM, cell propagation, and neovascularization [18–21]. Figure 1 shows the pathogenesis of TIs.

The natural curative procedure for torn tendons is complicated and medical results vary depending on the tendon tissue’s anatomical position. Three overlapping stages compose the healing of the wound process: the wound healing procedure, the proliferation stage, and the tissue remodeling stage [23]. Red blood cells invade the injured region during the inflammatory phase, followed by white blood cells and platelets vital GFs. Tendon cells, often referred to as tenocytes, attracted to the injured area are enhanced to proliferate, particularly in the epitenon [23]. Several cytokines are secreted by inflamed cells to enhance tendon repair, drive cell immigration, and promote angiogenesis. Included among these cytokines are insulin-like growth factor 1 (IGF-1), platelet-derived growth factor (PDGF), TGF-β, and basic FGF [24–26]. Under the direction of macrophages and tenocytes, the second phase, referred to as the propagative or restoration phase, is marked by a substantial quantity of artificial activity. Changing from phagocytic to reparative, macrophages release GFs [19,27]. In the third and final phase, called the remodeling stage, collagen I production assumes a central role. This stage begins 1–2 months after the damage and lasts over a year. The healed tissue has a similar look to a scar. Due to a rise in the content of water and a reduction in the amount and collagen quality, the biomechanical characteristics of the healed tissue can never be restored in full [19]. Due to the propensity of tendons to create adhesions, ruptures might happen later, resulting in a loss of load capacity sometimes noticed [28,29]. As a result of the absence of cells as well as GFs’ limited activity, the procedure by which torn tendons recover is often prolonged and feeble in the early stages [5,30]. Additionally, the mechanical characteristics of the repaired tendon were only 70% of those of the tendon before injury [31,32]. The creation of novel therapies is approaching. In this article, we will look at the research improvements and successes of numerous preclinical studies during the last decade from several perspectives.

## Epidemiology and etiology of tendon injury

The laceration is the most prevalent cause of flexor TI, with the greatest prevalence in those aged 20–29 years and a greater occurrence in men than in women. 25% of acute traumatic flexor TI are work-related, occurring most often in creation and removal (44%), preparing food and serving (14%), and shipping as well as material movement (12%) industries. Sports-related Achilles TIs are common, with around 50% of all sports-related wounds involving the Achilles tendon (AT). Among these injuries, approximately 75% of Achilles tendon ruptures occur in males aged 30–49. The primary cause of these injuries is sports participation [33,34]. Biopsies obtained after the operation have shown degenerative alterations in the majority of ruptured Achilles tendons4, indicating that AT ruptures may be categorized as acute trauma to persistently deteriorated tendons. Rotator cuff problems are the most prevalent cause of shoulder impairment, especially in the elderly [35]. 13% of adults in their 50s, 25% of individuals in their 60s, and 50% of those in their 80s had rotator cuff tears of complete thickness. The pathophysiology of rotator cuff tears is likely complex, with age-related deterioration variations [36] and micro/macro trauma playing a role. In addition to smoking, age, hypercholesterolemia, as well as family history have been identified as risk factors for rotator cuff tears [35]. It is important to understand that flexor and rotator cuff TI are intra-synovial and do not recover on their own, but injuries to the AT are extra-synovial and may and do result in fibrous tissue production. Because the surrounding climate and mechanisms of TI differ significantly between these [34] tendinopathic situations, the purpose of the study and models must be structured in light of these differences to create clinically existing findings that might ultimately be transformed into clinical practice.

## Mechanisms of tendon injury

**Figure 2.**
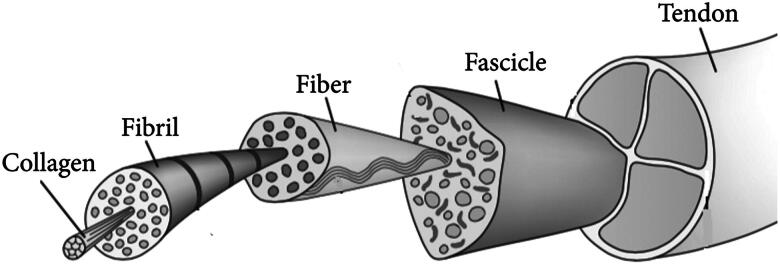
The hierarchical structure of tendons [37].

Tendons consist of a highly structured structure that permits the transfer of enormous stresses between bone and muscle throughout regular events. The structure is dependent on a tightly controlled interaction between the action of particular cell forms and the composition of ECM (Figure 2).

As tendons transfer stresses between bone and muscle through repetitive motion, they become prone to acute and chronic damage [38]. Several damage mechanisms may result in tendon rupture or tendinopathy, and the damage can be triggered by an amalgamation of acute and chronic trauma. Tendon ruptures, for instance, might take place in the context of laceration or acute overload, but are frequently the result of intrinsic disease [38] Sports participation is the most prevalent cause of AT fracture; however, biopsies have revealed deteriorating alterations in the majority of torn Achilles tendons [33,34,39]. Consequently, some writers classify this form of damage as acute trauma of persistently deteriorated tendons [38].

Along with injury caused by stressors that exceed physiological limitations, for instance, laceration or rupture, recurrent microtrauma inside physiological parameters may generate tendinopathy and raise the risk of chronic and acute damage. Loading that is repeated may be harmful to tendon structure because the healing system has a shorter time to mend microtraumas before successive pressures [40–42]. Microtears may also be caused by nonuniform force output and muscle activation, which leads to nonuniform tendon stress. This suggests that both load amount and distribution are crucial in determining the cause of tendon damage [40,41]. Hypoxia, oxidative stress, ischemia impairment, activation of apoptosis, and generation of cytokines that cause inflammation are among the theories offered to explain the origin of tendinopathy [43–48]. Tendinopathy may be caused, for instance, by relaxation and subsequent production of free radicals after ischemia produced by maximum tensile tension [43]. In instances of tendinopathy, the antioxidant enzyme peroxiredoxin [1], which is found in human tenocytes, is upregulated [44]. In addition, localized hypoxia may contribute to degeneration by preventing the maintenance of adequate ATP levels [45].

## Treatment of tendon injuries

The first treatment choices for acute and chronic TIs vary. Chronic TI therapy focuses mostly on reducing pain with anti-inflammatory medications, either local or systemic, whereas acute TI management tries to heal ruptured tendons using surgical procedures [3,49,50].

A mixture of modest rest and/or therapeutic interference is often sufficient for the cure of mild TI. A surgical procedure is the last resort cure for the ailment of the tendon. The success of reconstructive surgery depends on the type and location of the injury [3]. There are several suture methods, for instance, the restoration of 4 cross-locks, the U-shaped restoration, the Kessler type four-strand repair, etc. U-shaped and four-strand cross-lock restoration techniques could be superior [51]. Despite this, following tendon surgery, over 40% of surgical patients continue to have problems and poor functioning [52–55].

**Figure 3.**
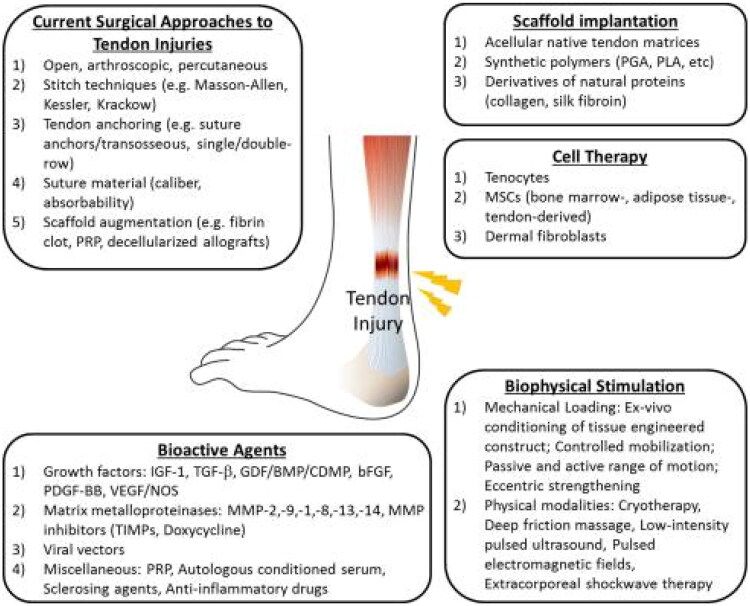
Illustration showing the strategies for tendon repairs. In brief, biophysical stimulation and surgical approaches are practiced in clinical settings. While the other approaches are currently in the preclinical or translational phase. Reproduced with permission from [56].

Faced with such a circumstance, it is important to discover innovative treatments. Several treatments, including medications, GT (gene therapy), GFs, SCT, PRP (platelet-rich plasma) treatment, and TE (tissue engineering), have been studied to assist the healing process. Figure 3 illustrates the various clinical and preclinical approaches to tendon repairs.

## Oral medication

For decades, oral non‐steroidal anti‐inflammatory drugs (NSAIDs) have been routinely utilized to alleviate tendon overuse-related pain. Recently, gels or patches for the local administration of NSAIDs have been promoted. Thirty-seven randomized clinical studies and systematic reviews of NSAIDs for the curing of tendinopathy were discovered *via* our search of the literature. Only 17 of these trials [57–73] were placebo-controlled. Generally, the data demonstrate that both local and oral NSAIDs are useful in providing short-term relief from tendinopathy-related pain (7–14 days). Only 3 of the 17 assessed trials [58,63,64] found no effect with NSAIDs. Oral and topical NSAIDs seem to be beneficial for treating acute shoulder tendonitis/bursitis [66–69,74]. One research has also shown the effectiveness of Naprosyn sodium in alleviating shoulder discomfort of longer duration [68]. In a randomized, double-blind, placebo-controlled research, Naprosyn sodium was more efficient than a placebo but not as useful as a corticosteroid injection in the curing of shoulder tendonitis/bursitis at 4 weeks. Individuals with symptoms that lasted longer and were more severe and more likely to respond poorly to corticosteroid injections and/or oral NSAIDs.

The effectiveness of NSAIDs in curing AT or lateral epicondylitis does not seem to be as great [58,63,75,76]. The only research that looked at the long-term efficiency of NSAIDs for the healing of lateral epicondylitis found no change between the placebo and naproxen-treated group at a 1-year follow-up [63]. A comprehensive evaluation of the use of NSAIDs for lateral epicondylitis revealed findings supporting the use of local NSAIDs with a reduction in pain over the short term (2 weeks) [75].

However, there are limited data available to support or challenge the long-term effectiveness of oral or topical NSAIDs. In addition, prolonged use of NSAIDs increases the likelihood of experiencing cardiovascular, renal, and gastrointestinal complications [77]. Overall, a low dosage of NSAIDs appears to be a viable option for treating acute pain resulting from excessive use of tendons, particularly in the shoulder [78]. There is insufficient evidence to definitively prove that NSAIDs are advantageous for the extended treatment of chronic tendinopathy.

## Physical therapy

**Figure 4.**
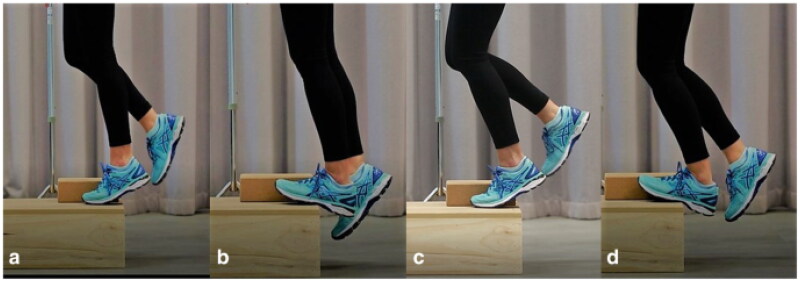
Eccentric calves. (a, b) Start and finish of the straight-knee exercise. (c, d) Start and finish of the bent-knee exercise [79].

In recent years, eccentric strengthening programs have been suggested for the cure of tendinopathy (Figure 4a–c) [79–82]. A previous study of the literature uncovered 16 controlled clinical studies and organized reviews examining this method. The control group in one of these experiments got no therapy [83]. At 4 months, the eccentric strengthening group performed better than the ‘wait and see’ group. Different clinical studies have assessed eccentric boosting when matched to other therapy methods [81,83–91]. For the cure of Achilles and Patellar tendonitis in recreational athletes [81,86], a 12-week program of eccentric boosting exercises outperformed a standard concentric enhancing regimen. In the AT trial, 82% of patients assigned to the eccentric strengthening program reported a reduction in pain, compared to 36% of those assigned to the concentric training group. Ultrasound and magnetic resonance imaging of the AT before and after a 12-week eccentric exercise regimen revealed weakening and normalization of tendon structure [92,93]. Intriguingly, eccentric strengthening yielded superior improvements in midsubstance AT than in insertional tendinopathy [94].

Also effective in the cure of lateral epicondylitis [95] has been eccentric strengthening programs. 92 individuals with lateral epicondylitis were randomized to a regular physical treatment routine with or without an eccentric bolstering program in well-designed research [95]. In comparison to the control group, the eccentric strengthening group indicated substantial improvements in pain, strength, and function. Patients allocated getting stretched and icing singly, or made longer and icing with concentric boosting did not differ in a comparable trial [88]. A literature review on eccentric strength training as a therapy for tendinopathy has been published [96]. This study indicated that there is minimal proof to substantiate the use of eccentric exercise above all other therapies, for instance, stretching, concentric exercise, splinting, ultrasound, and massage due to the diverse findings of the present research.

There are several modalities accessible to physical therapists, and it is impossible to determine which method or collection of treatments will be used. While a normal surgeon of orthopedic is not engaged in prescribing these therapies, it is helpful to be aware of the therapy options available and their efficacy. Iontophoresis, ultrasound, phonophoresis, and low-level lasers are FDA-approved treatments for tendinopathy; however, the 434-MHz hyperthermia instrument is not. A comprehensive mobilization and strengthening program for rotator cuff tendonitis has produced favorable results [97]. A total of 125 individuals were randomly allocated to either arthroscopic acromioplasty, a structured and monitored rotator cuff strengthening program, or a sham laser therapy in one research [98]. At 6 months, both the acromioplasty and physical therapeutic groups had lower pain levels than the placebo group. Pain levels did not vary between the two therapy groups. One more randomized clinical research allocated 52 individuals with shoulder impingement to manual physical treatment along with a strengthening and stretching program or strengthening and stretching alone [97]. The research found that including manual therapy improved pain levels and strength at the follow-up.

Low-level laser therapy (LLLT) has been intensively researched, with variable outcomes. Two of the 14 randomized controlled studies testing LLLT were rejected owing to insufficient controls. Five research [99,100] found that LLLT improved tendinopathy treatment when compared to placebo LLLT, whereas seven studies found no difference [101].

Other physical treatment approaches have not been explored as thoroughly, although the literature shows comparable mixed findings. Iontophoresis and phonophoresis are methods of delivering drugs locally that use ionizing current or ultrasound. Corticosteroids and NSAIDs are often utilized in conjunction with these methods. Only six well-controlled trials were found, and four of them showed no improvement above the controls [102]. Transverse friction massage has also been utilized to cure tendinopathy, although just three research [103] have looked at it. None of these researchers found that deep friction massage was superior to other physical treatment techniques. Deep friction massage was shown to have no advantage over other therapies in a Cochrane evaluation. Therapeutic ultrasonography is another kind of physical therapy that is often utilized in the cure of tendinopathy. On this topic, eight well-controlled studies and systematic reviews have been discovered [104].

## Gene therapy

Despite current technological advancements, restoration and renewal of tendon tissue remained a significant obstacle [105]. Barriers made of recycled synthetic biomaterials, blockers, and GT are among the techniques used by surgeons and researchers over the last several decades to expedite tendon healing and avoid tendon adhesion [106–110]. Among these treatments, GT is regarded as the most auspicious [111].

In GT, a non-native nucleic acid (for instance RNA or DNA) is introduced into a particular cell or tissue to cure a condition. Due to their negative charge and substantial size, such compounds need the transfer of gene vectors [112].

When a tendon is injured, critical genes for collagen synthesis are not stimulated in time. This is the underlying idea of GT for tendon recovery. In addition, following damage, genes that produce exaggerated scarring throughout tendon recovery may be upregulated, resulting in an imbalanced tendon-curing procedure. For more than a couple of years, the goal of GT has been to attain early curing strength with the least amount of adhesion surrounding the tendon following direct final reconstructive surgery by restoring this equilibrium [113].

Gene transfer techniques may be utilized to boost tendon curing because therapeutic gene arrangements can continuously enhance the curing reaction and come back to the tendon’s pre-injury function to the greatest extent achievable. Because of greater knowledge of physiology, tendon biology, disease pathogenesis, and healing mechanisms, GT’s benefits have been investigated [114].

Continuous expression of genes has the benefit of increasing endogenous gene components in target tissues, for instance, transcription factors and signal molecules [3]. Related to stem cell treatment, the incidence of immunological reactions is lower with GT [115]. GT may provide incessant local protein synthesis and secretion. This distinctive characteristic enables the limitation of protein supply related to short half-lives to be circumvented and for the occurrence of bioactivity signals in tissue locations to be controlled. GT is thus a potential technique for tendon redevelopment [105].

**Figure 5.**
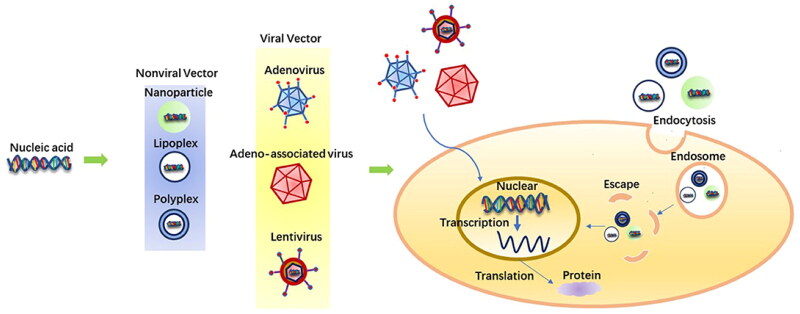
Distinct vectors in GT and gene transport procedures [116].

As depicted in Figure 5, GT is comprised of three important components: the gene transporter, the target gene, and the target cell. Genes often have a high negative charge density and a high molecular weight, therefore cell penetrability is restricted and GT is restricted [117,118]. Additionally, consideration must be given to the safe and efficient transport of nucleic acids into tendon tissues. Several vectors, containing viral vectors, nanospheres, and other transport methods, have been created in the deliberation of biocompatibility and the enhancement of the efficacy of GT [119–122].

An important criterion is the choice of gene transfer vectors for the healing of tendon tissue. The gene delivery vector has a significant influence on the effectiveness of GT [123,124]. Together viral and non-viral vectors have been examined for use in GT. In general terms, Viral vectors are efficient for transduction genes, although virus implantation mutation and quality management pose safety risks [112,125,126]. Even though non-viral vectors are largely harmless, there is a serious need to increase their transfection effectiveness and transgene appearance for clinical studies [127].

Several viral vectors, containing adenovirus (ADV), lentivirus, and adeno-associated virus (AAV) have been used to heal TI in animals [128–130]. As a common viral vector, ADV can transduce several separating and non-separating cells, resulting in stable but temporary gene expression. ADV transporter is the most common carrier used in clinical studies [131,132]. In 2003, AV formed the foundation for China’s first commercially authorized GT medication. Shenzhen GenTech’s altered ADV transporter Geneticine, which encodes the p53 tumor inhibitor gene, has been authorized for the cure of neck and head tumors. Due to a dearth of published data on clinical results, the clinical results linked with this treatment remain contentious [133,134]. Even though ADV is often used in clinical studies, there are several basic drawbacks. First, viral capsid proteins stimulate a robust immunological reaction, and second, the existence of the CAR (coxsackie and ADV receptor) on the cell membrane is required for cell transduction, causing preferable hepatocyte transduction after intravenous administration. Eventually, biological activity inside the body is constrained by time [135,136].

**Figure 6.**
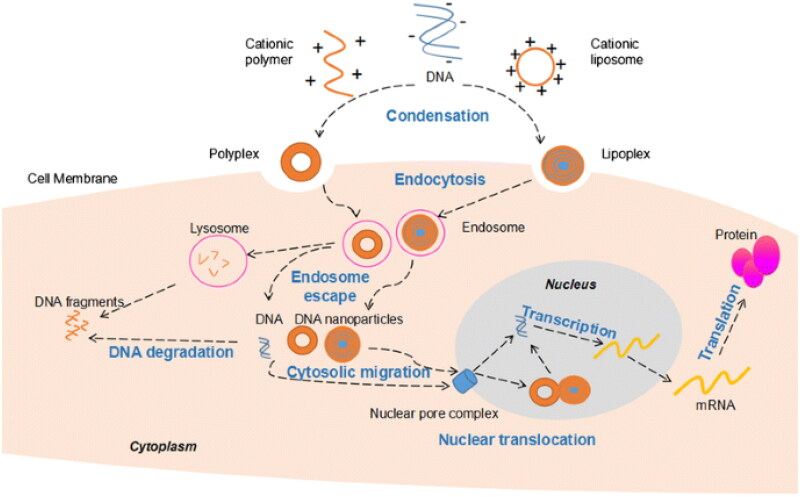
The basic mechanism of non-viral gene delivery [138].

Because of the negative adverse effects of viral vectors, non-viral vectors have gained increasing interest due to their low immunogenicity, safety, chemical design flexibility, high gene capabilities, and consistency. Moreover, non-viral vectors may be made quickly and extensively modified chemically. Moreover, the non-viral vector technology is not constrained by the size of the injected gene molecule [112]. Plasmid DNA (pDNA) is a type of double-stranded DNA, circular DNA that imitates independently from chromosomal DNA. pDNA may be simply designed to activate one or more genes, based on the intended use. pDNA has various benefits above viral distribution techniques, containing the capacity of plasmids to carry important genes [137]. It has been shown that PLGA poly(lactic-co-glycolic acid) nanospheres successfully distribute plasmids to cultivated muscle cells and wounded tendons. Nanospheres maintained a significant level of transgenes in tendons for a minimum of 6 weeks, with almost no tissue response seen. It might be a potential non-viral GT vector (Figure 6) [139]. Because of their subcellular framework, nanospheres can effectively improve gene-targeted distribution while also protecting gene sequences from deterioration, this makes it easier for them to get into the cell *via* endocytosis, access the cytoplasmic compartment, and allow the enclosed materials to be released for a long time, to overcome the cell membrane’s absorption barrier [140–142]. Additionally, polymers and cationic lipids may be employed as non-viral DNA vectors to generate lipoplex and polyplex [143,144]. As delivery vectors, several normal and artificial polymers have both been used effectively. Because naturally present polymers are separated from animal, plant, or human tissues, vehicles typically generated from these polymers have the benefit of mimicking the essential structural and/or biochemical features of ECM owing to their natural origin. Consequently, polymers that happen naturally benefit from low immunogenicity, relatively enormous amounts, and simple processing. Collagen and fibrin are instances of these polymers [145,146]. Scaffolds and hydrogels may be created in a gentle way to incorporate genetic vectors and cells that are more sensitive. Artificial polymers provide the discrete benefits of repeatability and regulated manufacture, allowing for accurate operation [146–148]. Furthermore, nuclear targeting of non-viral vectors with SV40 DNA-targeting sequences (DTS) may boost transfection efficiency . DTS induces nuclear entrance by incorporating plasmid DNA binding nuclear localization signals to transcription factors and directing its entry into the nucleus [112].

Each vector has intrinsic benefits and drawbacks; however, the assemblage of biomaterial transmitters may assist in compensating for the deficiencies. To accommodate for the absence of viral vector ADV, a few research have employed Surface coating and/or hydrogel encapsulation to protect ADV from host immune reactions [105]. Some studies have shown the possibility of combining biomaterials for instance collagen and fibrin with physical approaches to cure musculoskeletal damage [149–153].

Biomaterial system incorporation that emerges in gene transport tactics is fundamental to the vast majority of multiple sclerosis (MS) TE procedures and plays a function in the transport system of both viral and non-viral vectors [105,154]. Mechanical design, cell assistance, and biochemical signal control are all provided by these biomaterials. To accomplish this objective, biomaterial architectures have been constructed to resemble natural ECM [105].

GT may result in sustained local protein synthesis and secretion. This permits one to circumvent the constraints of protein transport caused by its short half-life and enables the manipulation of the timing of bioactive cues at a tissue location [105]. Therefore, GT is a viable option for MS tissue rejuvenation, but there are still crucial considerations to be made regarding the transport of nucleic acids to MS tissues safely and efficiently. To solve this, several administration routes, vector classes, and biomaterial carriers are being developed. GT may often be administered (1) directly *in vivo* or (2) indirectly *ex vivo*. In the indirect method, cells are transformed or altered *in vitro* and then re-administered to the patient. This method has the benefit of being able to manage the genetic mutation of specific cell populations safely, but it involves substantial cell acquisition and manipulation. In the direct method, the technical difficulty of manipulating cells is reduced, however, control over cellular targeting is limited and dosage. To accomplish cell-specific targeting and treating of genetic payloads, the direct method necessitates vector and/or carrier engineering design [105]. Table 1 summarizes some of the recent GT studies from the last couple of years.

**Table 1.** An overview of recent gene therapies for tendon healing.

Target gene	Function on tendon	Study type	Reference
COX	The COX inhibition has the potential to convert M1 to M2.	*In vivo*	[155]
IKKβ	*In vivo* inhibition of the KKβ/NF-B pathway could be effective in the treatment of RCT.	*In vivo*	[156]
Smad3	The delivery method acts as an excellent antiadhesion barrier, potentially lowering inflammation.	*In vivo*	[157]
ITGA9	Tenascin-C enhances tendon rejuvenation through STSC migration mediated by ITGA9.	*In vitro*	[158]
CLK2 DYRK1A	SM04755 decreases inflammation and improves tenocyte differentiation by suppressing CLK2 and DYRK1A	*In vitro*	[159]
Collagen III	PolyDMAEA-siRNA polyexes outperform PEI-siRNA polyexes in terms of efficiency.	*In vitro*	[160]
Flightless I	Flii has the potential to inhibit human tenocyte propagation and migration.	*In vitro*	[161]
Scleraxis	Scx raises tenomodulin levels.	*In vitro*	[162]

## An overview of stem cell-based tendon restoration strategies

### Tendon restoration with stem cells

Stem cells may be categorized depending on their ability for lineage differentiation. Pluripotent stem cells (PSCs), for instance, embryonic stem cells (ESCs) and induced pluripotent stem cells (iPSCs), can develop into any kind of body cell. Multipotent stem cells, such as MSCs can differentiate into a wide range of cell forms, although their differentiation capacity is restricted in comparison to pluripotent cells.

ESCs constitute a single source of cells that might be utilized to take the place of any additional type of cell in the body that has been lost because of injury or illness; hence, they have a significant amount of potential in renewing medicine, comprising tendon repair and redevelopment [163]. Chen et al. revealed that human embryonic stem cells (hESCs) differentiated into MSCs (hESC-MSCs), assimilated into a fibrin gel [164] or onto a knitted silk-collagen sponge scaffold [165], and embedded into a rat tendon fault developed tendon structural and mechanical possessions. Although these are encouraging outcomes, it has to be determined if this treatment technique also aids patients with chronic TI, for example, tendinopathy [166].

iPSCs are pluripotent stem cells (PSCs) that may be produced automatically from adult cells by adding four certain genes that encode transcription factors that turn adult somatic cells into PSCs [166–168]. Cells produced from iPSCs exhibit low immunogenicity upon implantation [168], this is one of the reasons why iPSCs have become popular cell resources for tissue restoration. Xu et al. [169] were the first to show that iPSC-derived neural crest stem cells (NCSCs) adjourned in a fibrin gel and transplanted into a patellar tendon window deformity in rats dramatically accelerated tendon repair in comparison to rats cured with only fibrin gel. During the 4-week experimental investigation [169], the embedding of iPSC-NCSCs resulted in cured damaged tissue that revealed no ectopic cartilage or bone development. iPSCs may be a viable basis for stem cell-based tendon restoration therapies, although long-term efficacy testing is essential. In addition, a new *in vitro* study employing equine iPSCs revealed that, unlike equestrian ESCs, when cultivated in 3D collagen gels, equine iPSCs were not able to create synthetic tendons [170]. In 2D differentiation tests, equine iPSCs displayed tendon-associated proteins and genes. The regulatory mechanism necessary for iPSCs to acquire the restorative and reformative potential for tendon healing must be further investigated.

MSCs can develop into several cell types of connective tissue, containing tenocytes. MSCs may be formed from many types of tissue, including bone marrow, tendon, and adipose tissue. Several animal research investigations have revealed that MSC-based cell-based therapies promote tendon healing (studied in [3,171]). MSCs from peripheral blood are evolving cell resources for tendon recovery, MSCs from umbilical cord blood (UCB-MSC), and periodontal ligament cells (PDLCs). Injection of MSCs from peripheral blood enhanced histologic characteristics of a damaged tendon in a sheep model of tendinopathy caused by collagenase [172]. Allogeneic UCB-MSCs inserted into spontaneously developing superficial digital flexor tendon tendinitis resulted in increased effectiveness and power, along with enhanced curing, as determined by imaging of ultrasound [173]. In the rabbit model, UCB-MSCs have also been demonstrated to boost tendon-bone curing after anterior cruciate ligament restoration [174]. Intravenous administration of UCB-MSCs into the bone tunnel-tendon implant interface enhanced the bone-tendon interface’s histological appearance [174]. Moreover, ancestor cells/periodontal ligament-derived stems acquired from individuals receiving orthodontic management accelerated the repair of a full-thickness AT fault development lesion, with comparable effectiveness to AT-derived cells [175]. Although several stem cell forms have demonstrated effectiveness in animal models, a standard management methodology might be necessary to analyze and relate curative outcomes and find the most auspicious stem cell choice for a particular kind of tendon healing.

Various research studies use just one kind of stem cell. Interestingly, the combination of several stem cells in a single injection improved the therapeutic impact. Coculture of bone marrow mesenchymal stem cells (BM-MSCs) with tendon stem/progenitor cells (TSPCs) at a ratio of 1:1 boosted tenogenic differentiation as shown by elevated tenogenic markers and collagen I expression [176]. Embedding of cell sheets produced from TSPCs and BM-MSCs were injected into a rat patellar tendon window deformity improved tendon repair significantly, especially in contrast to deformities cured solely with BM-MSCs or TSPCs [176].

Amniotic epithelial stem cells (AECs) possess distinctive regenerative characteristics that render them a potentially fruitful pathway in the realm of tendon repair [177]. These cells, which originate from the amniotic membrane, exhibit multipotent properties that allow them to undergo differentiation into cells resembling tendons and promote the regeneration of tissues. Anti-inflammatory (AEC) properties include immunomodulatory effects and the secretion of numerous GFs that stimulate tissue regeneration and healing [178]. The capacity of AECs to augment tendon repair through the stimulation of collagen synthesis, promotion of angiogenesis, and modulation of the ECM has been demonstrated in numerous studies. Ethical considerations and non-invasive procurement render them appealing contenders for regenerative therapies in the context of TIs. Leveraging the regenerative capabilities of AECs presents considerable potential for the progression of tendon repair methodologies and the enhancement of clinical results [179,180].

Whereas several stem cell forms have been shown to improve tendon repair, there have been reports of safety issues and unwanted effects. As presented by bone marrow stem cells (BMSCs) [181] and adipose-derived stem cells (ADSCs) [182], there is a danger of ectopic bone production and tumor development under certain conditions. After transplantation, there is a danger of teratoma development in both ESCs and iPSCs [183]. Gaining stem cells for instance TSPCs might also result in morbidity at the donor location. Therefore, it is difficult to acquire a significant quantity of stem cells of excellent quality for therapeutic applications for instance TSPCs. In addition, stem cell cultivation conditions need special consideration. TSPCs cultivated under hypoxic settings (5% O_2_) displayed increased cell propagation and stem cell indicator expression Oct-4, Nanog, and SSEA-4 compared to TSPCs grown under normoxic conditions (20% O_2_) [184]. Furthermore, TSPCs cultured in hypoxia, implanted onto a decellularized tendon matrix, and embedded in nude rats subcutaneously formed more substantial tendon-like structures [184], implying that a hypoxic environment could be perfectly adapted for preserving the stemness of TSPCs in culture.

Recent advancements notwithstanding, a few clinical evidences exist addressing the healing efficiency of utilizing stem cells to cure TI and disorders. A latest comprehensive review [185] revealed four clinical trials [53,186–188] employing BM- [187,188] as well as allogenic atypical squamous cells (ASCs) [53,186] to cure tendon diseases (patellar tendinopathy, lateral epicondylar tendinopathy, and rotator cuff tears are all examples of tendinopathies) [53,186–188]. These four trials discovered that stem cell therapy increased tendon recovery, as measured by imaging, functional results, and pain ratings [53,186–188]. However, only one of the trials [187] included a control group, and none of the four investigations were blinded, creating a significant possibility of biased outcomes. The findings should thus be treated with care [185]. Several fundamental and translational investigations are required before stem cell-based treatments for TIs and illnesses may be suggested for normal clinical use.

In addition, many difficult problems remain unanswered, such as: what kind of stem cells are the best candidate for tendon healing therapies? [18]. Does the heterogeneous population of MSCs include subpopulations of cells that may result in a more advantageous result [33], and which damages or disorders need stem cell implantation? [34] To what degree may exosomes produced from stem cells mimic the healing impact of stem cells in tendon damage and/or ailment? Furthermore, given there are several biological distinctions between acute and chronic TI, as well as the healing processes after these wounds [38], specialized stem cell treatment techniques may need to be customized for each kind of tendon damage. Studies examining these issues and concerns could not only progress basic knowledge of the processes underpinning the positions of stem cells in tendon healing and rejuvenation, but they also demonstrate scientific basis and viability for stem cell-based strategies for instance stem cell-derived biologics, stem cell insertion, and designed to induce endogenous stem localization. In preclinical and clinical investigations, it is crucial to create uniform methods and procedures for collection, pretreatment, amplification, distribution, and follow-up care.

### Tissue engineering

TE is a multifaceted method intended to stimulate tissue replacement, repair, or restoration. TE is the process of creating functional tissue utilizing a mix of cells, scaffolds, and bioactive chemicals [189]. In the early stage, the torn tissue will be substituted by tendons from other regions of the body, tendons taken from some other person, tendons from some of the other species, or synthetic tendons. These implants include autograft, xenograft, allograft, and artificial grafts [190]. Donor site difficulties are the main downside of autografts. Xenografts and allografts are constrained by the accessibility of contributor tissues and the possibility of immunological rejection and pathogen spread [3]. The use of xenograft still wants to lower its high contagion incidence, according to a clinical investigation [191]. The downsides of early synthetic grafts include early re-tear, a loss in mechanical strength over time, inadequate ingrowth of tissue, and implant wreckage deposition [192]. Despite the improved function of the current generation of grafts, the deployment of these synthetic implants remains contentious since clinical studies have not yet reached a united and definitive assumption [193].

Due to the issues, there has been a growing interest in tissue-engineered tendon preparation during the last decade. The objective of TE is to offer a secure and efficient alternative for biologically damaged tissue [194]. Two or all three of the following components are present in tissue-engineered grafts: cells, biomolecules, and biomaterials/scaffolds [195]. A technique of TE implants that offers a physical setting to manage the therapeutic and redevelopment of wounded tissue is the development of an engineered scaffold. Figure 3 depicts scaffold preparation and use. Scaffolds serve primarily to provide a physical setting that regulates the curing and renewal of wounded tissues [196].

To stimulate tissue healing and restoration, stem cells are often implanted onto constructed scaffolds. Tendon stem cells can be collected from various sources, including tendon tissue itself, bone marrow, adipose tissue, and other mesenchymal tissues [197]. However, the procurement of tendon stem cells directly from tendon tissue can be challenging due to the invasive nature of the procedure and the limited availability of tendon tissue [7]. Alternatively, bone marrow and adipose tissue are common sources for isolating mesenchymal stem cells, including tendon stem cells. These stem cells can be obtained through minimally invasive procedures such as bone marrow aspiration or liposuction [198]. Additionally, tendon stem cells have also been identified within the tendon sheath, which surrounds the tendon and can be accessed through less invasive procedures [199]. They possess anti-inflammatory properties, including lowering tissue inflammation and stimulating tissue regeneration by secreting a vast variety of trophic substances. This may explain their widespread usage in ligament and tendon TE [200–202]. Secretory bodies, exosomes, and PRP are examples of cell secretory materials that may be transplanted into engineered tissues to enhance the therapeutic impact [203–205].

Scaffolds act as a temporary 3-D construct with an adequate microenvironment that replicates the structure and function of the underlying tendon and reduces progressively as the repaired tissue develops. Porous structures with varying pore diameters must be biocompatible with little or no immunological rejection by the body; the rate of degradation must be equivalent to the renewal rate of the tendon tissue, and the breakdown products must be harmless [206]. The following qualities should be present in optimal biomaterials for scaffold construction: improved mechanical strength during the process of regenerating tissue [207]; superior biocompatibility with neighboring tissues; biodegradability with a variable rate of breakdown; excellent biofunctionality to promote cell propagation, secretion of ECM, differentiation, and tissue development; flexibility in processing to build desirable structures; and wettability and hydrophilicity to support cell proliferation, differentiation, ECM secretion, and tissue formation [207,208].

Natural substances are widely employed in TE due to their ease of derivatization, improved biocompatibility as well as biodegradability, favorable cell adhesion, and other biochemical signals [209]. They can be categorized as either polysaccharide substances (hyaluronic acid, alginate, chitosan) or protein biomaterials (fibrin, silk, and collagen) [210]. Most of these materials have disadvantages, such as weak mechanical characteristics, uncontrollable deterioration time, and significant batch-to-batch variance, which limits their clinical applicability [211]. Due to their excellent biomechanical qualities, biodegradability, as well as commercial availability, synthetic biomaterials for instance polyglycolic acid (PGA) [212], polylactic acid (PLA) [213], PLGA, and polycaprolactone (PCL) family of polymers have been frequently employed in TE [214]. Compared to scaffolds made of natural materials, the mechanical properties of artificial polymer scaffolds were superior. However, they lack cell-binding ligands and biological signals, and they do not exhibit optimum adhesion, infiltration, growth, or tissue development [215].

Stem cells have the potential to propagate and synthesize active paracrine substances, stimulate immunological control and hence tendon renewal, and differentiate into tendon cells. Hence, stem cells have significant applicability value in tendon restoration through TE [216]. Bi et al. [216] identified TSPCs in mouse and human tendons, and Schneider et al. [216] validated their presence in numerous tendons and ligaments from various species. TSPCs are clonogenic multipotent adult stem cells with the potential for multilineage differentiation, and surface stemness markers [217]. The two primary forms of PSCs, ESCs, and iPSCs, have a high capacity for propagation and differentiation. ESCs can differentiate into all 3 germ layers’ tissues and proliferate [218]. In comparison to MSCs, ESCs have a higher ability to survive and migrate through the tendon, as well as the potential to provide adequate cell numbers due to their unparalleled propagation capacity. Tenocytes and fibroblasts are the two most prevalent forms of unipotent cells. They have the advantage of not producing teratomas, unlike stem cells [219]. *In vivo*, they lack the ability for self-renewal, though they can proliferate *in vitro* to a limited extent. Inadequate functions resulting from phenotype drift with growing generations are also a cause for concern [220].

All aspects of tendon TE have made significant strides, and the field exhibits potential future research opportunities. Numerous biomaterials comprised of natural and artificial polymers have been utilized in scaffolds and have demonstrated superior biodegradability, biocompatibility, and biomechanical properties [221]. Various scaffolds made up of compositions and structures resembling the native tendon’s hierarchical architecture have been fabricated, and cell products, various cell types, and other critical components have been included to aid in tendon rejuvenation and repair [222]. Table 2 highlights some of the scaffolds that have been used in preclinical applications and those provided on the market and used for clinical applications. Though promising development has been made in tendon TE in terms of the underlying mechanisms and *in vitro* or animal model experimental techniques, much more work is necessary to allow for future clinical use [56,239].

**Table 2.** Scaffolds that have been used in preclinical applications and those provided on the market and used for clinical applications.

Scaffolds	Preclinical applications	Clinical applications	References
Synthetic polymer scaffolds	Tailorable mechanical properties Controlled degradation rates Supports cell adhesion and proliferation	GORE-TEX^®^ Soft Tissue Patch TenoGlide^®^ Synthetic Polymer Scaffold	[223–225]
Hyaluronic acid-based scaffolds	Enhances cell viability and proliferation Provides a hydrated environment conducive to tissue repair Exhibits anti-inflammatory properties	HYAFF^®^ Scaffolds HYALOFAST^®^ Hyaluronic Acid-Based Scaffold	[226–228]
Collagen-based scaffolds	Encourages cell adhesion and proliferation Supports tendon tissue formation and alignment Enhances biomechanical properties of the repaired tendon	BioFiber^®^ Scaffold Integra^®^ Dermal Regeneration Template	[229,230]
Silk-based scaffolds	Biocompatible and biodegradable Offers tunable mechanical properties Supports cell attachment and proliferation	SeriScaffold^®^ Silk-Based Scaffold Ortho-ATI^®^ Silk-Based Scaffold	[231–233]
Electrospun nanofiber scaffolds	Mimics the native extracellular matrix structure Promotes cell attachment and migration Supports tissue regeneration and remodeling	Nanofiber-based products for tendon repair (e.g., OrthADAPT^®^ Bioresorbable Nanofiber Matrix)	[234,235]
Decellularized tissue scaffolds	Retains native tissue architecture and composition Provides a natural scaffold for cell infiltration and tissue regeneration	GraftJacket^®^ Regenerative Tissue Matrix ArthroFlex^®^ Decellularized Tendon Allograft	[236–238]

### Growth factor therapy

Since tendon damage stimulates the creation and release of various GFs throughout the healing process, recombinant GFs continue to be studied as possible tendon-healing treatments [56]. Although no human research investigations have been published on this technology, *in vivo* and *in vitro* research have proved its effectiveness and prospective influence on the field. The emphasis of research on the role of GFs in tendon renovation has been on IGF-1, vascular endothelial growth factor (VEGF), TGF, PDGF, and FGF [5,240].

The injured site may be treated with GFs by local injection, surgery, coated sutures, or scaffolding substances implantation. Even though there has been less research on coated sutures and scaffolding approaches, it has been claimed that GFs are removed from the site of damage at a slower pace than with local injection. Four investigations on the function of GFs have revealed their capacity to control the differential expression of collagens and stimulate cell propagation [3]. The administration of several GFs resulted in reduced dosage needs and greater potency compared to the administration of individual GFs [241].

The interaction between various GFs through the curative process helps to clarify the reported effectiveness of autologous GF concentrates, for instance, PRP, utilized in therapeutic settings. Due to the poorly defined nature of the concentrate and the wide range of technologies used to create PRP, studies on the use of autologous GFs remain conflicting [242]. This heterogeneity produces unpredictability in the GF and cytokine composition of the combination, making it difficult to examine and compare the findings [243].

There is some evidence that GF treatment promotes tendon renewal. Nevertheless, it remains doubtful that a single GF could rebuild normal tendons. The utilization of growth-factor combination treatment, which includes the delivery of various GFs in a temporal and geographical distribution that resembles normal physiology, is a prospective field for future study. This ‘growth-factor cocktail’ may be able to influence curing *via* several pathways and at different times post-injury, allowing for more precise regulation of the therapeutic process. Moreover, research into biomaterials that could supply GFs in a spatially and temporally specified way has shown promise for emulating the healing cascade [244].

## Conclusion

The tendon is a unique tissue with distinct framework, features, and mechanical properties. Destruction of this important connective tissue may result in excruciating pain and impairment. Improving tendon repair is hence an important need. In the realm of tendon curing and restoration, several study topics exist. There have been some advances in gene therapy, stem cell treatment, and other fields, but they are not yet clinically applicable. Although several previous research has concentrated on tendon repair, there are still numerous questions to be answered concerning this intricate procedure.

Current research uncovered a clear correlation between tumors and the healing of wounds. From cancer research, we may be able to get a greater comprehension of tendon repair and so discover techniques to speed tendon recovery. For instance, there are indications that the temporary shutdown of the apoptotic regulation mechanism might speed up normal healing.

## Data Availability

The data that support the findings of this study are available from the corresponding author (WL) upon reasonable request.
